# Fucoidan Alleviates Porcine Epidemic Diarrhea Virus-Induced Intestinal Damage in Piglets by Enhancing Antioxidant Capacity and Modulating Arginine Metabolism

**DOI:** 10.3390/ani15071001

**Published:** 2025-03-30

**Authors:** Qian Zhang, Maojing Wang, Zhonghua Li, Di Zhao, Yongqing Hou, Tao Wu

**Affiliations:** Hubei Key Laboratory of Animal Nutrition and Feed Science, Wuhan Polytechnic University, Wuhan 430023, China; zhangqian870305@126.com (Q.Z.); 13263987813@163.com (M.W.); yuxincangqiong@163.com (Z.L.); zhaodi24@163.com (D.Z.); houyq@aliyun.com (Y.H.)

**Keywords:** fucoidan, piglets, porcine epidemic diarrhea virus, intestinal damage, antioxidant, arginine metabolism

## Abstract

Porcine epidemic diarrhea virus (PEDV) causes severe intestinal damage, posing significant threats to the swine industry. Fucoidan (FUC), a biologically active compound, exhibits antiviral activity against multiple viruses. This study aimed to investigate the protective effects of FUC on PEDV-induced intestinal injury in piglets and explore its underlying mechanisms. A total of 28 healthy crossbred piglets were randomly allocated into four experimental groups using a 2 × 2 factorial design: (1) a control group, (2) an FUC group, (3) a PEDV group, and (4) an FUC+PEDV group. From day 4 to day 10, the piglets in the FUC and FUC+PEDV groups were orally administered fucoidan at a dosage of 20 mg/kg body weight (BW) each day. On day 8, the piglets in the PEDV and FUC+PEDV groups were orally administered PEDV at a dose of 3 × 10^5.5^ TCID_50_. The results show that FUC supplementation significantly decreased plasma DAO activity (*p* < 0.05) and increased the villus height, villus area, as well as the villus height/crypt depth (*p* < 0.05) in the intestine when compared to the PEDV-infected piglets. This indicates that FUC could alleviate the disruption of intestinal morphology and function caused by PEDV infection. FUC enhanced the antioxidant capacity of the piglets by increasing SOD and GSH-Px activity. Transcriptional profiling combined with quantitative analysis revealed that FUC regulates immune responses, substance transport, and arginine metabolism. Notably, FUC downregulated arginase 1 expression, which may redirect arginine toward nitric oxide synthesis, thereby establishing an antiviral state in the host. These findings highlight the potential application of FUC as a natural agent for mitigating PEDV-induced intestinal damage and improving gut health. Additionally, monitoring the health status of piglets is necessary when FUC is applied in practical applications.

## Simple Summary

This study aimed to investigate the protective effects of FUC on PEDV-induced intestinal injury in piglets and to explore its underlying mechanisms. The results show that FUC supplementation could alleviate the disruption of intestinal morphology and function caused by PEDV infection. FUC enhanced the antioxidant capacity of the piglets. Transcriptional profiling combined with quantitative analysis revealed that FUC regulates immune responses, substance transport, and arginine metabolism. Notably, FUC downregulated arginase 1 expression, suggesting a redirection of arginine toward nitric oxide synthesis, thereby placing the host in an antiviral state. These findings highlight the potential application of FUC as a natural agent for mitigating PEDV-induced intestinal damage and improving gut health.

## 1. Introduction

The animal intestinal tract plays a pivotal role in nutrient absorption and barrier function, which are essential for maintaining adequate nutrition. Beyond its role in nutrient regulation, the intestinal tract also serves as a critical barrier, preventing pathogen invasion and preserving intestinal integrity [1]. In practice, numerous factors can contribute to intestinal damage in piglets, among which the porcine epidemic diarrhea virus (PEDV) is a prominent causative agent. PEDV causes porcine epidemic diarrhea (PED), which is an acute, highly contagious enteric disease. This virus exhibits high pathogenicity and can infect pigs of all ages and breeds, with piglets being the most severely affected, often presenting with pronounced clinical symptoms, including vomiting, diarrhea, and lethargy [2]. PEDV primarily targets intestinal epithelial cells, causing rapid cell lysis, necrosis, and villous atrophy [3]. Additionally, the enzymatic activity and content of damaged cells are significantly diminished, leading to a compromise of the gut’s nutrient absorption capacity [4]. Despite widespread vaccination, the infection rate of PEDV has risen significantly due to the highly mutated rates of amino acids within the neutralizing epitopes of the spike gene [5,6]. This poses a substantial threat to the swine industry nationwide.

Polysaccharides derived from animals, plants, and microorganisms are natural products known for their ability to regulate various physiological functions. They are characterized by their safety, low toxicity, and minimal side effects [7]. Fucoidan (FUC), a sulfated polysaccharide primarily composed of L-fucose and sulfate groups, also contains trace amounts of xylose, mannose, arabinose, galactose, and glucuronic acid [8]. FUC is predominantly found in the cell walls and intercellular spaces of brown algae, such as kelp and wakame [9]. FUC does not cause significant toxicity within reasonable dosage ranges. Recent studies have demonstrated that FUC exhibits a wide range of biological activities, including anticoagulant, antibacterial, antiviral, antitumor, and immunomodulatory effects [10,11]. FUC modulates the intestinal mucosal barrier, thereby stabilizing the microecological environment and mitigating inflammatory responses [12]. FUC promotes intestinal health by regulating gut microbiota, repairing the intestinal mucosa, enhancing digestive enzyme activity, and increasing the number of goblet cells [13]. Moreover, it attenuates pro-inflammatory responses, such as IL-6 and TNF-α, in porcine intestinal epithelial cells stimulated by E. coli through the NF-κB signaling pathway, while also reducing bacterial adhesion and invasion [14]. Numerous studies have demonstrated that both naturally extracted and synthetic sulfated polysaccharides exhibit inhibitory activity against a variety of DNA and RNA viruses in vitro and in vivo [15]. Research indicates that FUC can inhibit the entry of SARS-CoV-2 into host cells by binding to the spike glycoprotein [16,17]. Additionally, FUC has been shown to effectively block influenza A virus infection in vitro, with low toxicity and minimal propensity for inducing viral resistance [18]. Although FUC has been shown to protect the intestinal tract and exhibit antiviral effects, there is a lack of research on its ability to defend against viral infections in the intestines of pigs and/or other animal species. Therefore, we conducted this study to evaluate the effects of FUC on intestinal injury induced by PEDV in piglets.

## 2. Materials and Methods

### 2.1. Experimental Materials and Diets

Fucoidan (purity > 85%) was purchased from Shanghai Yuanye Bio-Technology Co., Ltd. (Shanghai, China). A PEDV strain (Yunnan Province strain 8) was provided by the Hubei Key Laboratory of Animal Nutrition and Feed Science at Wuhan Polytechnic University, Wuhan, China.

### 2.2. Animals and Experiment Design

A total of 28 healthy 7-day-old crossbred piglets (initial body weight: 2.49 ± 0.38 kg) were selected for this experiment. All the piglets were negative for PEDV by the experiment, which was confirmed by real-time PCR. They were randomly allocated into four experimental groups using a 2 × 2 factorial design: (1) a control group, (2) an FUC group, (3) a PEDV group, and (4) an FUC+PEDV group. The experimental period lasted for 11 days, with the first three days allocated for adaptation. From day 4 to day 10, piglets in the FUC and FUC+PEDV groups were orally administered fucoidan (dissolved in milk) at a dosage of 20 mg/kg body weight (BW) each day. The same volume of milk was provided to the piglets in the control and PEDV groups. On day 8, piglets in the PEDV and FUC+PEDV groups were orally administered PEDV at a dose of 3 × 10^5.5^ TCID_50_. This dosage was determined according to our previous study, with slight adjustments [19]. Piglets in the control and FUC groups received an equivalent DMEM solution. Fasting was initiated at 22:00 on day 10, with water and feed withdrawn for 8 h. At 06:00 on day 11, fasting body weights were recorded, followed by sequential oral administration of d-xylose (0.1 g/kg body weight) via gavage. Peripheral blood samples were collected from the jugular vein using EDTA-coated vacutainers. Terminal procedures commenced with intramuscular anesthesia (sodium pentobarbital, 50 mg/kg BW). Tissue samples were immediately snap-frozen in liquid nitrogen and stored at −80 °C for subsequent analysis.

Piglets were weighed on an empty stomach each morning on days 0, 4, 8, and 11, with weights recorded to calculate the average daily gain (ADG). Throughout the experimental period, diarrhea and morbidity were observed both before and after each feeding session, and fecal morphology was assessed using a standardized scoring system: normal, dry feces were scored as 0; feces in a pasty state were scored as 1; feces in a semi-liquid state were scored as 2; and feces in a liquid state were scored as 3.

### 2.3. Biochemical Measurements in Plasma

Blood samples were gently inverted several times to ensure thorough mixing of the blood with the anticoagulant and to prevent coagulation. Plasma and serum were subsequently separated by centrifugation. And the fresh plasma samples were immediately used for biochemical parameter analysis with a Hitachi 7100 Automatic Biochemical Analyzer (Hitachi, Tokyo, Japan).

### 2.4. Determination of D-Xylose and Diamine Oxidase Activity in Plasma

Colorimetric methods were employed to measure d-xylose content and diamine oxidase (DAO) activity in plasma using kits obtained from the Nanjing Jiancheng Bioengineering Institute (d-xylose: catalog number A035-1-1; DAO: catalog number A088-1-1). All assays were conducted in accordance with the manufacturer’s instructions.

### 2.5. Antioxidant Capacity in Serum and Intestinal Mucosa

The antioxidant enzymes and their associated products were analyzed using serum and intestinal mucosa samples from piglets. The activities and concentrations of glutathione peroxidase (GSH-Px), superoxide dismutase, total superoxide dismutase (T-SOD), myeloperoxidase (MPO), malondialdehyde (MDA), and hydrogen peroxide (H_2_O_2_) were measured using commercially available reagent kits from the Nanjing Jiancheng Institute of Bioengineering (Nanjing, China).

### 2.6. Intestinal Histomorphology

Briefly, 1 cm intestinal segments were fixed in 4% paraformaldehyde, dehydrated, and embedded in paraffin. Sections with a thickness of 6 µm were cut, deparaffinized with xylene, and stained with hematoxylin and eosin. Morphometric parameters, including the villus height (VH), crypt depth (CD), villus width (VW), and the ratio of the villus height to crypt depth (VH/CD), were measured using an Olympus BX41 microscope (Tokyo, Japan) and analyzed with the ImageProPlus 6.0 software (Media Cybernetics, Rockville, MD, USA).

### 2.7. Real-Time PCR

The total RNA was extracted from approximately 100 mg of intestinal tissue using RNAiso Plus (Takara, Dalian, China). RNA concentration was quantified using a NanoDrop^®^ ND-2000 UV-VIS spectrophotometer (Thermo Scientific, Wilmington, DE, USA), and RNA integrity was verified by 1% agarose gel electrophoresis. Complementary DNA (cDNA) was synthesized using the PrimeScript^®^ RT reagent Kit with gDNA Eraser (Takara, Dalian, China). Gene expression levels were analyzed by quantitative real-time PCR (qPCR) using SYBR^®^ Premix Ex Taq™ (Tli RNaseHPlus) (Takara, Dalian, China) on an Applied Biosystems 7500 Fast Real-Time PCR system (Applied Biosystems, Waltham, MA, USA). The ribosomal protein L19 (RPL19) gene served as the internal reference, and relative gene expressions were calculated and statistically analyzed using the 2^−ΔΔCt^ method. Primer sequences for the target genes are provided in Table 1.

### 2.8. Sequencing and Analysis of the Jejunal Transcriptome

Twelve jejunal samples from three treatment groups were selected for transcriptome analysis. The total RNA was extracted from the tissues using the Trizol reagent, and RNA quality was assessed to ensure purity and integrity. Eukaryotic mRNA was enriched using oligo (dT) magnetic beads and reverse-transcribed into cDNA. Sequencing libraries were constructed and quality-checked using an Agilent 2100 Bioanalyzer (Agilent Technologies, Santa Clara, CA, USA). High-throughput sequencing was performed on the Illumina Novaseq 6000 platform. Differentially expressed genes (DEGs) were identified with thresholds set at *q* value < 0.05 and fold change > 1.5. Functional annotation of the DEGs was conducted through Gene Ontology (GO) analysis using the GO database (http://geneontology.org/) (accessed on 23 January 2025) and pathway enrichment analysis using the Kyoto Encyclopedia of Genes and Genomes (KEGG) database (https://www.kegg.jp/) (accessed on 23 January 2025).

### 2.9. Statistical Analyses

Statistical analysis was performed using two-way ANOVA (2 × 2 factorial design) through the General Linear Model procedure in SPSS 26.0 (SPSS Inc., Chicago, IL, USA). The model included the main effects of dietary treatment (FUC-supplemented vs. FUC-free) and the challenge status (DMEM vs. PEDV), as well as their interaction term. Post hoc comparisons were conducted using Duncan’s test when significant interactions (*p* < 0.05) were detected. Data are expressed as means ± pooled standard errors of the mean (SEM), with statistical significance defined at *p* < 0.05.

## 3. Results

### 3.1. Growth Performance

As shown in Table 2, PEDV infection significantly reduced ADG and increased the diarrhea scores of the piglets (*p* < 0.05). However, FUC supplementation had no significant effect on ADG and diarrhea scores after PEDV infection.

### 3.2. The mRNA Levels of PEDV Genes in the Jejunum, Ileum, and Colon

The relative expression levels of PEDV M, N, and S mRNA are summarized in Table 3. PEDV infection significantly increased the relative expression of PEDV M, N, and S mRNA, and the supplementation of FUC resulted in a further increase in their expression levels (*p* < 0.05).

### 3.3. Plasma Biochemical Parameters

The effects of FUC on blood biochemical parameters in piglets infected with PEDV are shown in Table 4. Compared with the uninfected PEDV piglets, the PEDV-infected piglets had increased UREA concentrations (*p* < 0.05) and decreased concentrations of lactate dehydrogenase (LDH), total cholesterol (TC), high-density lipoprotein (HDL-C), and direct bilirubin (DB) in plasma (*p* < 0.05). FUC administration decreased plasma LDH, total bilirubin (TB), HDL-C, and DB (*p* < 0.05). There were significant interactive effects between PEDV and FUC on plasma TB and DB concentrations (*p* < 0.05).

### 3.4. Blood DAO Activity and D-Xylose Concentrations

Data on plasma DAO activity and d-xylose concentrations are shown in Table 5. Compared to the uninfected PEDV piglets, PEDV infection significantly decreased plasma d-xylose concentrations (*p* < 0.05). There were interactive effects between PEDV and FUC on DAO activity. Following PEDV infection, FUC supplementation significantly decreased plasma DAO activity (*p* < 0.05) when compared to the PEDV-infected piglets.

### 3.5. Intestinal Morphology

**Figure 1:**
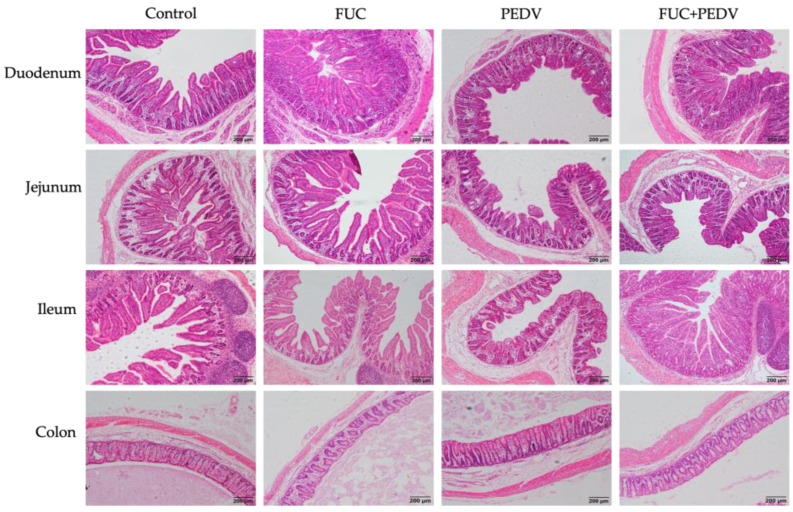
Histopathological structures of piglets’ duodenum, jejunum, ileum, and colon in the control, FUC, PEDV, and FUC+PEDV groups.

PEDV infection induced obvious villous atrophy in the small intestine, which improved with FUC supplementation (Figure 1). As shown in Table 6, compared to the uninfected PEDV piglets, the PEDV-infected piglets had a lower villus height, villus area, and villus height/crypt depth ratios in the small intestine and a lower villus width in the duodenum and jejunum. Additionally, these infected piglets showed a lower crypt depth in the jejunum and a higher crypt depth in the colon (*p* < 0.05). Piglets supplemented with FUC had higher villus height-to-crypt depth ratios in the small intestine and higher villus heights in the duodenum and jejunum (*p* < 0.05). Notably, there was an interaction between PEDV infection and FUC supplementation on the intestinal villus height, villus area, and villus height/crypt depth ratios. Specifically, the piglets in the FUC+PEDV groups showed a significantly higher villus height and villus area in the duodenum and ileum as well as a higher villus height/crypt depth in the duodenum (*p* < 0.05) compared to the PEDV groups.

### 3.6. Intestinal Antioxidant Capacity

The effects of FUC supplementation on the intestinal antioxidant capacity in the PEDV-infected piglets are shown in Table 7. Compared to the control group, FUC supplementation significantly reduced H_2_O_2_ levels in the jejunum, ileum, and colon as well as the MDA content in the ileum and MPO activity in the duodenum, jejunum, and colon (*p* < 0.05). PEDV infection resulted in lower H_2_O_2_ concentrations in the plasma, jejunum, and ileum as well as decreased T-SOD activities in plasma and GSH-Px activities in the ileum (*p* < 0.05). Additionally, MDA concentrations were elevated in the duodenum and colon (*p* < 0.05). Interaction effects between PEDV infection and FUC supplementation were observed, with the infected piglets receiving FUC exhibiting lower H_2_O_2_ levels in the jejunum, increased SOD activity (*p* < 0.05), reduced MDA concentrations in the ileum (*p* < 0.05), and enhanced GSH-Px activity alongside decreased MPO activity in the duodenum (*p* < 0.05).

### 3.7. Transcriptome Analysis

**Figure 2:**
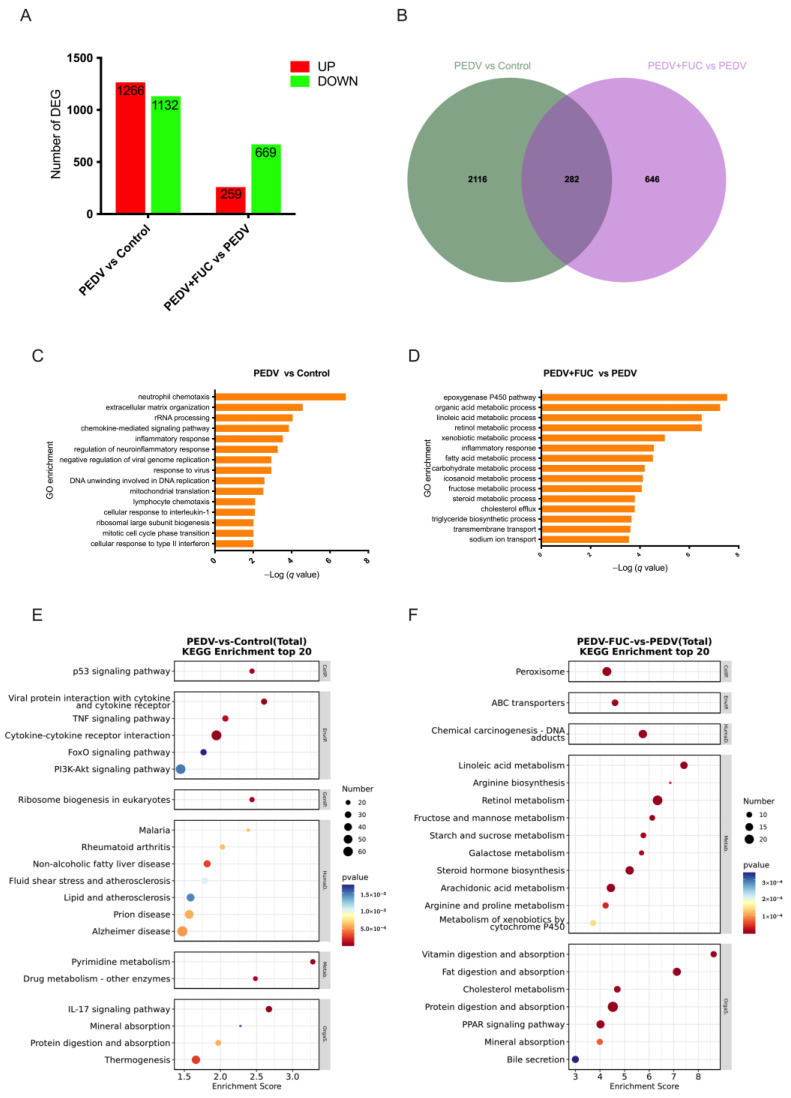
Transcriptomic analysis of PEDV-infected piglets treated with FUC in jejunum. (**A**) Overview of differentially expressed genes (DEGs; fold change > 1.5; q value < 0.05) in the jejunum. (**B**) Venn diagram showing overlapping DEGs between groups. (**C**,**D**) Biological process enrichment analysis of DEGs between different groups. (**E**,**F**) KEGG analysis of DEGs between different groups.

A total of 2398 and 928 DEGs were identified in the PEDV vs. control and PEDV+FUC vs. PEDV comparisons, respectively. And the number of overlapped genes between the PEDV vs. control and PEDV+FUC vs. PEDV comparisons was 282 (Figure 2A,B). To elucidate the biological processes associated with PEDV pathogenesis and FUC’s therapeutic effects, GO and KEGG analyses were systematically performed. The DEGs between the control and PEDV groups were predominantly enriched in immune response-associated biological processes, such as “neutrophil chemotaxis”, “chemokine-mediated signaling pathway”, “inflammatory response”, “regulation of neuroinflammatory response”, and “cellular response to type II interferon”, and virus-associated biological processes, such as “negative regulation of viral genome replication” and “response to virus” (Figure 2C). Similar findings were observed in the KEGG pathway analysis (Figure 2E). In addition, “mineral absorption”, “protein digestion and absorption”, “pyrimidine metabolism”, “p53 signaling pathway”, “PI3K-Akt signaling pathway”, and “FoxO signaling pathway” were also enriched for the DEGs between the control and PEDV groups. DEGs between the PEDV and PEDV+FUC groups were mostly enriched in metabolism-associated biological processes, especially lipid metabolism-associated biological processes, such as “linoleic acid metabolic process”, “fatty acid metabolic process”, “steroid metabolic process”, “cholesterol efflux”, and “triglyceride biosynthetic process” (Figure 2D). Substance transport processes, such as “transmembrane transport” and “sodium ion transport”, were also significantly enriched. Similar findings were observed in the KEGG pathway analysis (Figure 2F). Notably, arginine metabolisms, such as “arginine biosynthesis” and “arginine and proline metabolism”, were significantly enriched.

### 3.8. The Expression of Immune-Associated Genes upon PEDV Infection and FUC Supplementation in the Jejunum of Piglets

**Figure 3:**
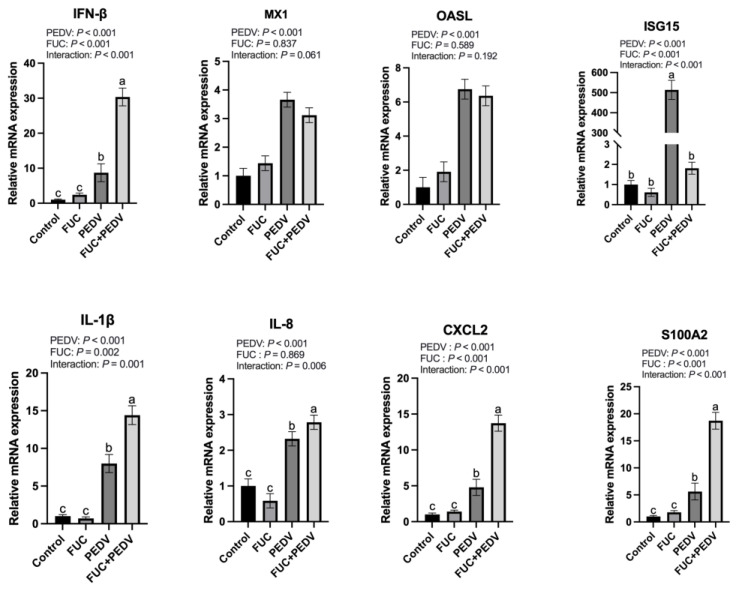
The mRNA expression of genes related to immune responses in the jejunum of piglets. MX1: Myxovirus Resistant 1; OASL: 2′-5′-Oligoadenylate Synthetase Like; ISG15: Interferon-Stimulated Gene 15; IFN-β: Interferon β; IL-1β: Interleukin-1β; CXCL2: Chemokine Ligand 2; S100A2: S100 Calcium-Binding Protein A2; IL-8: Interleukin-8. Values are means and pooled SEMs; *n* = 7. ^a,b,c^ Within a row, means with different superscripts differ; *p* < 0.05.

During viral infection, both antiviral-related genes and inflammatory cytokines were significantly upregulated (*p* < 0.05) (Figure 3). A significant interaction effect between PEDV infection and FUC supplementation was observed on mRNA expression levels of ISG15, IFN-β, IL-1β, CXCL2, S100A2, and IL-8 (*p* < 0.05). FUC supplementation further increased the expression of IFN-β, IL-1β, CXCL2, S100A2, and IL-8 while significantly downregulating ISG15 expression compared to PEDV infection alone.

### 3.9. The Expression of Transport- and Arginine Metabolism-Associated Genes upon PEDV Infection and FUC Supplementation in the Jejunum of Piglets

**Figure 4:**
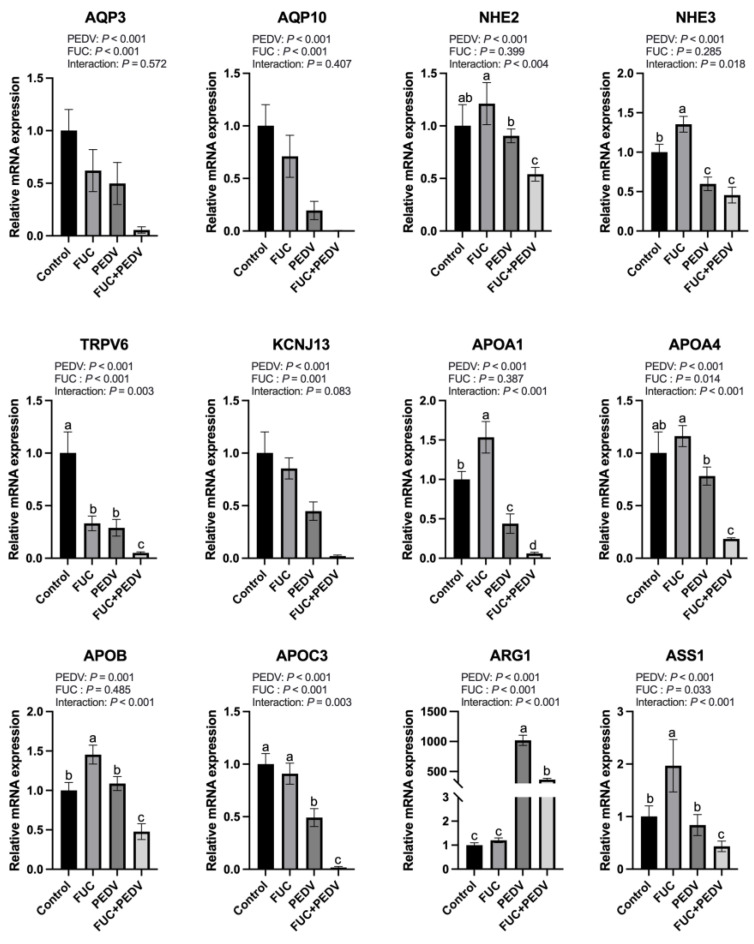
The mRNA expression of genes related to transport and arginine metabolisms in the jejunum of piglets. AQP3: Aquaporin 3; AQP10: Aquaporin 10; NHE2: Na+/H+ Exchanger 2; NHE3: Na+/H+ Exchanger 3; TRPV6: Transient Receptor Potential Cation Channel Subfamily V Member 6; KCNJ13: Potassium Inwardly Rectifying Channel Subfamily J Member 13; APOA1: Apolipoprotein A1; APOA4: Apolipoprotein A4; APOB: Apolipoprotein B; APOC3: ApolipoproteinC3; ARG1: Arginase 1; ASS1: Argininosuccinate Synthase 1. Values are means and pooled SEMs; *n* = 7. ^a,b,c,d^ Within a row, means with different superscripts differ; *p* < 0.05.

During PEDV infection, the mRNA expression levels of aquaporin genes (AQP3 and AQP10); sodium–hydrogen exchangers (NHE2 and NHE3); apolipoproteins (APOA1, APOA4, APOB, and APOC3); TRPV6; KCNJ13; and ASS1 were significantly downregulated (Figure 4), while the mRNA expression levels of ARG1 were significantly upregulated. Notably, with the exception of AQP3, AQP10, and KCNJ13, PEDV infection showed interaction effects with FUC supplementation on the transcriptional regulation of other investigated genes. Specifically, the addition of FUC caused a further reduction in mRNA expression levels of NHE2, TRPV6, APOA1, APOA4, APOB, APOC3, and ASS1 compared to PEDV infection alone, while FUC supplementation counteracts PEDV-induced ARG1 upregulation.

## 4. Discussion

Gastrointestinal health is a critical factor in animal performance, and plant-derived polysaccharides have emerged as an important source of bioactive compounds with significant pharmacological properties [20], among which FUC is a promising candidate for addressing gastrointestinal disorders, such as inflammatory bowel disease (IBD) [21,22]. In this study, we demonstrated that fucoidan can improve the morphology and structure of the intestine, repair the intestinal barrier, and enhance the antioxidant status in PEDV-infected pigs. Furthermore, transcriptomic analysis was employed to elucidate the regulatory mechanisms underlying these effects. This study highlights the potential of FUC as an effective strategy for PEDV prevention and control and provides a theoretical basis for its application in production practices.

Plasma biochemical indicators reflect an organism’s health status, particularly in terms of metabolism, immune regulation, and cellular function [23]. Key lipids such as TC and HDL-C indicate lipid metabolism efficiency [24]. Chronic infections, including hepatitis C virus and COVID-19, often reduce serum TC and HDL-C levels due to impaired liver function or systemic inflammation [25,26]. Similarly, patients with IBD exhibit lower TC and HDL levels compared to healthy individuals [27]. In this study, PEDV infection in piglets significantly decreased plasma TC and HDL levels. This indicates that PEDV infection may disrupt lipid metabolism, possibly due to systemic inflammation or oxidative stress caused by the virus. The elevated plasma urea levels observed in this study may reflect increased protein catabolism or impaired renal function [28]. However, FUC supplementation did not have any effect on plasma TC, HDL, and urea levels in the PEDV-infected piglets. Notably, FUC supplementation call-backed TB and DB levels, which were increased by PEDV infection in the plasma. This suggests that FUC supplementation may have a protective effect on liver function [29]. Although PEDV mainly targets enteric cells, severe infections can lead to the systemic circulation of viral particles, potentially infecting hepatocytes directly. Moreover, the oxidative stress induced by PEDV infection may increase reactive oxygen species production, overwhelming the liver’s antioxidant defenses and causing hepatocyte damage, which impairs liver function. In this study, FUC alleviated oxidative stress in the intestine, which may contribute to reducing hepatic injury and protecting liver function. From this perspective, FUC may play a protective role in mitigating PEDV-induced systemic effects.

This study demonstrates that PEDV infection compromises gut barrier function and small intestinal absorption in piglets, as evidenced by elevated plasma DAO activity and reduced d-xylose absorption, which aligns with our previous studies [19]. The intestinal dysfunction may have resulted from intestinal epithelial atrophy and desorption. In this study, we found that FUC mitigated the damage caused by PEDV infection. FUC has been shown to enhance small intestinal barrier function and restore cecal flora balance in weaned goat pups and has been demonstrated to activate the PI3K/Akt pathway to promote cell survival and proliferation, thereby facilitating tissue regeneration [12,30]. FUC’s anti-inflammatory, antioxidant, and immunomodulatory functions could also contribute to repairing intestinal damage [10,11]. In addition, FUCs act as prebiotics, selectively promoting the growth of beneficial bacteria, such as Lactobacillus and Bifidobacterium, while inhibiting pathogenic bacteria. A balanced gut microbiota reduces systemic inflammation, enhances nutrient absorption, and supports the repair of intestinal tissues [12]. However, inconsistent results have been reported, as highlighted by Bonnie Homer’s study, which found no significant improvement in intestinal morphology upon FUC supplementation [31,32]. This discrepancy may be attributed to several factors, including variations in the experimental conditions employed and the molecular characteristics of the FUCs. Studies have demonstrated that a low-molecular-weight FUC with a high sulfate content exhibits stronger bioactivity compared to its high-molecular-weight counterparts [33,34]. A low molecular weight allows for better absorption and interaction with cellular receptors, while a high sulfate content enhances bioavailability and pharmacokinetic properties. Overall, FUCs demonstrate repair effects on intestinal injury induced by PEDV infection.

Under normal physiological conditions, the body maintains a redox balance to prevent oxidative damage. However, this equilibrium is disrupted during pathogen invasion, leading to oxidative stress. PEDV infection alters antioxidant enzyme activities and increases peroxidation products in piglets’ blood and intestines, indicating a reduced antioxidant capacity and elevated oxidative stress. In this study, FUC supplementation showed positive effects by enhancing GSH-Px and T-SOD activities while reducing MDA levels. These findings indicate that FUCs alleviate PEDV-induced oxidative stress by improving the antioxidant capacity and protecting intestinal health. Similar findings were observed in other studies. Ryu et al. (2016) found that FUCs upregulate the expression of antioxidant enzymes, such as SOD, at the transcriptional level by activating Nrf2, a master regulator of antioxidant response [35]. In Caco-2 cells, FUC activated the Nrf2/HO-1 pathway to facilitate the antioxidant activities under oxidative stress through elevating the SOD and GSH levels [36]. The authors proposed that FUCs’ negatively charged sulfate groups directly interact with the positively charged regions on these enzymes, thus forming a protective layer around the enzyme and stabilizing their structure. In conclusion, FUC supplementation shows promise in mitigating oxidative stress, highlighting FUC as a potential natural adjunctive therapy for improving the antioxidant capacity during viral infections.

Following a virus infection, the host innate immune system is rapidly activated. Generally, IFNs prime neighboring cells via JAK-STAT pathways to upregulate ISGs, which establish an antiviral state to restrict viral replication and spread [37]. Concurrently, pro-inflammatory cytokines would be induced, recruiting mononuclear inflammatory cells to the infection site and eliminating viral particles [38,39]. However, dysregulated cytokine production would cause serious tissue damage [40]. In this study, PEDV infection significantly upregulated the expression of multiple antiviral and inflammation-related factors in the jejunum, which aligns with our previous findings [19], while FUC supplementation further enhanced the jejunal expression of IFNs and other pro-inflammatory cytokines in PEDV-infected piglets. Notably, ISG15—an interferon-stimulated gene—displayed no concomitant elevation in the PEDV+FUC group despite increased IFN levels. This phenomenon may be attributed to the transient early-phase response characteristics of ISG15, which typically exhibits peak expression during initial viral invasion followed by time-dependent attenuation [41]. Moreover, emerging evidence suggests that ISG15 exerts both anti- and pro-viral effects. The free, extracellular ISG15 has been proposed to implicate in the hyperinflammation seen in severe COVID-19 [42]. Moreover, ISG15 has been shown to regulate cellular processes that include autophagy and metabolism [43]. As for PEDV, we have shown that ISG15 was highly induced upon infection. Its downregulation may contribute to PEDV replication, as we noticed in this study. However, its specific role remains to be investigated. FUC is considered to have anti-inflammatory effects; therefore, the upregulation of inflammatory cytokines observed by FUC supplementation was unexpected. Possible reasons for this are not yet certain and may be related to the dosage of fucoidan, the sampling time, and the condition of the animals. This could be also have been caused by the upregulation of PEDV levels. Furthermore, it is well recognized that mucosal immunity, especially IgA levels, is crucial for managing PEDV infection [39]. Previous studies have shown that FUCs can act as a mucosal adjuvant [44]. Therefore, in subsequent research, it is warranted to examine the influence of FUC on mucosal immune responses during PEDV infection. This will contribute significantly to our understanding of the protective mechanisms by which FUC defends against PEDV in infected piglets. Together, FUC supplementation could modulate immune responses in the PEDV-infected piglets, and attention should be paid to its effects on viral proliferation and inflammatory cytokine expression in practical applications.

The transcriptomic results reveal that the addition of FUC modulates intestinal transport processes. We performed real-time PCR to detect the expression levels of genes related to ion transport, water channels, and lipid transport. A significant downregulation of these genes was observed during PEDV infection. These changes likely exacerbated diarrhea, nutrient malabsorption, and barrier dysfunction, facilitating viral dissemination [45,46,47]. However, FUC supplementation further reduced their expression. This result puzzles us greatly, because FUC showed an improving effect on intestinal function and morphology, which seems to contradict the downregulation of these genes. On the other hand, this may be an adaptive response of the host to the infection, reducing the virus’s utilization of host resources, and FUC enhances this response. Additionally, FUC may compensate for the function of these genes through other pathways, such as activating alternative transport mechanisms or promoting cell proliferation. Moreover, changes in the gut microbiota and their metabolism may indirectly promote intestinal repair. For example, studies have shown that fucose exhibits a beneficial effect on the restoration of the richness and diversity of the gut microbiota community and the beneficial bacterium Muribaculaceae. Concurrently, significant improvements were observed in the structural damage of the intestinal mucosa [48]. Overall, FUC could regulate the expression of genes involved in ion transport, water channels, and lipid transport, which requires careful risk–benefit evaluations in PEDV-infected piglets.

Importantly, we found that FUC supplementation modulated arginine metabolism during PEDV infection. In this study, PEDV infection significantly upregulated the expression of ARG1 while downregulating ASS1 expression. Notably, FUC administration mitigated the PEDV-induced upregulation of ARG1. As ARG1 catalyzes the conversion of arginine to ornithine and urea, its increased activity reduces arginine availability [49]. ASS1 plays a critical role in arginine synthesis within the urea cycle; its downregulation hinders arginine regeneration, exacerbating intracellular arginine depletion. The resulting scarcity of arginine limits the substrate for inducible nitric oxide synthase (iNOS), consequently diminishing nitric oxide (NO) production—a key antiviral molecule [50]. By downregulating ARG1 expression, FUC supplementation may inhibit the metabolic diversion of arginine toward ornithine and urea, redirecting arginine flux toward NO synthesis. This metabolic adjustment could enhance host antiviral defense mechanisms via NO-mediated immune regulation. This is supported by another study which shows that FUC could enhance nitric oxide synthase phosphorylation, thereby affecting NO production [51]. Further studies will be needed to explore the specific mechanisms by which FUC impacts arginine metabolism and to confirm its role in PEDV infection.

## 5. Conclusions

In summary, the supplementation of FUC demonstrated significant benefits in mitigating PEDV-induced pathologies. Specifically, FUC improved intestinal morphology and function, enhanced the antioxidant capacity of piglets, and modulated immune responses within the intestine. Moreover, FUC intervention regulated transport processes and arginine metabolism in PEDV-infected piglets.

However, to fully realize the therapeutic potential of FUC, future research should prioritize optimizing its structural properties and delivery parameters. Specifically, efforts should be directed toward standardizing FUC formulations based on molecular weights and sulfate contents to ensure consistent efficacy. Investigating dose–response relationships and time-dependent effects is also a critical step toward maximizing FUC’s therapeutic benefits. Additionally, elucidating the underlying mechanisms by which FUC interacts with gut epithelial cells and the gut microbiota will provide deeper insights into its modes of action. By addressing these factors, researchers can better harness the potential of FUC as a natural agent for mitigating intestinal damage and improving gut health.

## Figures and Tables

**Table 1:** Primer sequences used for qPCR analysis.

Gene	Forward (5′-3′)	Reverse (5′-3′)	Size (bp)	References
RPL19	AACTCCCGTCAGCAGATCC	AGTACCCTTCCGCTTACCG	147	Present study
PEDV-M	TCCCGTTGATGAGGTGAT	AGGATGCTGAAAGCGAAAA	231	Present study
PEDV-N	TTGGTGGTAATGTGGCTGTTC	TGGTTTCACGCTTGTTCTTCTT	180	Present study
PEDV-S	CTCTCTGGTACAGGCAGCAC	GCTCACGTAGAGTCAAGGCA	131	Present study
IFN-β	AGCAGATCTTCGGCATTCTC	GTCATCCATCTGCCCATCAA	101	[19]
MX1	AGTGCGGCTGTTTACCAAG	TTCACAAACCCTGGCAACTC	150	[19]
OASL	GGCACCCCTGTTTTCCTCT	AGCACCGCTTTTGGATGG	139	[19]
ISG15	AGCATGGTCCTGTTGATGGTG	CAGAAATGGTCAGCTTGCACG	164	Present study
IL-1β	CAACGTGCAGTCTATGGAGT	GAGGTGCTGATGTACCAGTTG	372	Present study
CXCL2	CGGAAGTCATAGCCACTCTCAA	CAGTAGCCAGTAAGTTTCCTCCATC	143	Present study
S100A2	TAGGGCAGGTTTGGTTTCCTT	GGGGACTGGACATCTTGGAT	148	Present study
IL-8	TTCGATGCCAGTGCATAAATA	CTGTACAACCTTCTGCACCCA	176	Present study
AQP3	AAGCTGTCCCAAGTAAAGCACAA	GCCCTACTTCCTGTTTCACCAC	251	Present study
AQP10	GGGCGTTATACTAGCCATCTAC	CCAACTGCACCAAGGAGTAA	134	Present study
NHE2	CAGCGACTCAGACCCAGACA	CCACCTCGTTCTTCCATTCC	128	Present study
NHE3	AAGTACGTGAAGGCCAACATCTC	TTCTCCTTGACCTTGTTCTCGTC	341	Present study
TRPV6	AGGAGCTGGTGAGCCTCAAGT	GGGGTCAGTTTGGTTGTTGG	146	Present study
KCNJ13	ATGGATGTGTCGCTGGTCTTT	CACAACTGCTTGCCTTTACGAG	141	Present study
APOA1	CCTTGGCTGTGCTCTTCCTC	ACGGTGGCAAAATCCTTCAC	100	Present study
APOA4	ACCCAGCAGCTCAACACTCTC	GAGTCCTTGGTCAGGCGTTC	122	Present study
APOB	GGGATGATGGCACAGGTTACA	TGACGTGGACTTGGTGCTTT	123	Present study
APOC3	CTAACCAGCGTGAAGGAGTC	CAGAAGTCGGTGAACTTGCC	116	Present study
ARG1	GGCTGGTCTGCTTGAGAAAC	ATCGCCATACTGTGGTCTCC	216	Present study
ASS1	CCCTCACTTTGCCCATCTCT	CCCTACCCTTCCGTTTGCT	163	Present study

**Table 2:** Effects of FUC supplementation on growth performance after PEDV infection.

Items	−PEDV		+PEDV		SEM	*p* Vaule		
	−FUC	+FUC	−FUC	+FUC		PEDV	FUC	PEDV × FUC
ADG (Kg/d)	0.087	0.093	0.061	0.052	0.007	0.03	0.901	0.589
Diarrhea score	0.000	0.014	0.086	0.143	0.002	0.004	0.304	0.535

PEDV, porcine epidemic diarrhea virus; FUC, fucoidan; ADG, average daily gain; SEM, standard error of the mean.

**Table 3:** Effects of FUC supplementation on the relative mRNA expression levels of PEDV in the small intestine of piglets infected with PEDV.

Items	PEDV	FUC+PEDV	SEM	*p* Value
Jejunum				
PEDV-M	1.000 ^b^	0.809	0.070	0.182
PEDV-N	1.000 ^b^	2.696 ^a^	0.264	0.000
PEDV-S	1.000 ^b^	3.381 ^a^	0.377	0.001
Ileum				
PEDV-M	1.000 ^b^	1.558 ^a^	0.108	0.004
PEDV-N	1.000 ^b^	2.099 ^a^	0.168	0.000
PEDV-S	1.000 ^b^	2.012 ^a^	0.176	0.003
Colon				
PEDV-M	1.000 ^b^	2.497 ^a^	0.244	0.000
PEDV-N	1.000 ^b^	1.971 ^a^	0.173	0.001
PEDV-S	1.000 ^b^	1.689 ^a^	0.122	0.001

PEDV, porcine epidemic diarrhea virus; FUC, fucoidan; PEDV-M, the M gene of PEDV; PEDV-N, the N gene of PEDV; PEDV-S, the S gene of PEDV; SEM, standard error of the mean. ^a,b^ Within a row, means with different superscripts differ; *p* < 0.05.

**Table 4:** The effects of FUC supplementation on plasma biochemicals in PEDV-infected piglets.

Iterms	−PEDV		+PEDV		SEM	*p* Value		
	−FUC	+FUC	−FUC	+FUC		PEDV	FUC	PEDV × FUC
TB (μmol/L)	3.564 ^a^	3.781 ^a^	4.320 ^a^	1.813 ^b^	0.267	0.107	0.004	0.001
TP (g/L)	47.557	47.257	47.314	52.400	1.847	0.105	0.113	0.076
ALB (g/L)	28.229	28.714	28.914	29.814	1.137	0.457	0.563	0.862
ALT (U/L)	41.957	45.200	49.471	50.143	2.425	0.110	0.606	0.735
ALP (U/L)	375.602	371.166	347.514	320.450	17.668	0.152	0.560	0.675
TC (mmol/L)	3.264	2.697	2.466	2.471	0.130	0.002	0.070	0.065
TG (mmol/L)	0.559	0.534	0.601	0.593	0.028	0.258	0.706	0.855
Glu (mmol/L)	5.426	5.691	5.201	5.407	0.238	0.427	0.461	0.925
CREA (μmol/L)	73.071	76.814	70.900	73.414	3.459	0.590	0.545	0.905
UREA (mmol/L)	3.333	4.274	8.139	8.726	0.567	<0.001	0.250	0.787
LDL-C (mmol/L)	1.264	1.040	1.006	1.044	0.054	0.107	0.234	0.097
HDL-C (mmol/L)	1.434	1.164	1.007	0.850	0.063	<0.001	0.003	0.388
LDH (U/L)	421.000	416.600	414.971	342.014	17.012	0.030	0.037	0.062
CK (U/L)	217.473	218.200	224.543	150.700	13.048	0.160	0.092	0.086
DB (μmol/L)	1.461 ^b^	1.812 ^ab^	2.126 ^a^	0.480 ^c^	0.142	0.031	<0.001	<0.001

Values are means and pooled SEMs; *n* = 7. ^a,b,c^ Within a row, means with different superscripts differ; *p* < 0.05. TB: total bilirubin; TP: total protein; ALB: albumin; ALT: alanine amino transferase; ALP: alkaline phosphatase; TC: total cholesterol; TG: triglyceride; GLU: glucose; CREA: creatinine; UREA: urea; HDL: high-density lipoprotein; LDL: low-density lipoprotein; LDH: lactate dehydrogenase; CK: creatine kinase; DB: direct bilirubin.

**Table 5:** The effects of FUC supplementation on DAO activity and d-xylose concentration in PEDV-infected piglets.

Items	−PEDV		+PEDV		SEM	*p* Value		
	−FUC	+FUC	−FUC	+FUC		PEDV	FUC	PEDV × FUC
DAO (U/L)	16.56 ^c^	25.69 ^b^	29.15 ^a^	20.35 ^bc^	1.387	0.110	0.941	<0.001
d-xylose (mmol/L)	1.45	1.43	1.26	0.97	0.054	0.001	0.070	0.106

Values are means and pooled SEMs; *n* = 7. ^a,b,c^ Within a row, means with different superscripts differ; *p* < 0.05. DAO: diamine oxidase.

**Table 6:** The effects of FUC supplementation on intestinal morphology in PEDV-infected piglets.

Items	−PEDV		+PEDV		SEM	*p* Value		
	−FUC	+FUC	−FUC	+FUC		PEDV	FUC	PEDV × FUC
Duodenum								
VH (μm)	360.908 ^a^	355.606 ^ab^	249.086 ^d^	319.961 ^c^	10.645	0.000	0.022	0.009
CD (μm)	158.726	154.45916	157.01286	147.83488	2.817	0.474	0.252	0.672
VW (μm)	110.78343	111.11917	94.985	101.03155	1.914	0.000	0.291	0.344
VA (μm^2^)	40,924.675 ^a^	35,171.610 ^b^	23,976.708 ^d^	29,286.451 ^c^	1469.229	0.000	0.899	0.004
VH/CD	2.268 ^a^	2.317 ^a^	1.590 ^b^	2.171 ^a^	0.065	0.000	0.000	0.001
Jejunum								
VH (μm)	371.570	383.556	215.493	236.209	15.455	0.000	0.122	0.672
CD (μm)	139.394	139.591	132.195	126.422	1.961	0.008	0.434	0.403
VW (μm)	97.912	96.059	91.078	86.727	1.151	0.000	0.075	0.461
VA (μm^2^)	34,976.145	33,560.680	20,664.134	22,078.758	1371.049	0.000	1.000	0.251
VH/CD	2.670	2.756	1.628	1.876	0.101	0.000	0.041	0.307
Ileum								
VH (μm)	354.070 ^a^	353.723 ^a^	222.773 ^b^	326.387 ^a^	12.900	0.000	0.004	0.004
CD (μm)	128.167	113.220	126.508	133.883	3.550	0.179	0.587	0.117
VW (μm)	100.346	97.891	91.363	99.488	1.904	0.338	0.460	0.174
VA (μm^2^)	35,681.037 ^a^	31,296.865 ^ab^	20,144.727 ^d^	29,651.764 ^c^	1398.454	0.000	0.180	0.001
VH/CD	2.813	3.163	1.786	2.443	0.125	0.000	0.005	0.361
Colon								
CD (μm)	232.782	212.569	271.902	239.249	5.321	0.000	0.001	0.395

Values are means and pooled SEMs; *n* = 7. ^a,b,c,d^ Within a row, means with different superscripts differ; *p* < 0.05. VH: villus height; CD: crypt depth; VA: villus area; VW: villus width.

**Table 7:** The effects of FUC supplementation on the intestinal antioxidant capacity in PEDV-infected piglets.

Items	−PEDV		−PEDV		SEM	*p* Value		
	−FUC	+FUC	−FUC	+FUC		PEDV	FUC	PEDV × FUC
H_2_O_2_ (mmol/L; mmol/mg protein)								
plasma	32.036	29.730	23.475	18.953	1.575	0.001	0.201	0.673
duodenum	14.036	12.748	14.016	12.263	0.472	0.793	0.122	0.809
jejunum	10.175 ^a^	9.879 ^a^	9.444 ^a^	6.867 ^b^	0.360	0.002	0.015	0.047
ileum	16.9 ^a^	13.021 ^b^	14.082 ^ab^	9.111 ^c^	0.683	0.004	0.000	0.427
colon	8.722	7.713	8.437	7.085	0.228	0.273	0.008	0.677
T-SOD (U/mL; U/mg protein)								
plasma	84.550	84.143	79.201	82.189	0.633	0.002	0.222	0.112
duodenum	240.66 ^a^	220.99 ^ab^	212.56 ^b^	233.89 ^ab^	3.92	0.289	0.906	0.007
jejunum	183.145 ^a^	143.881 ^ab^	141.3 ^b^	175.654 ^ab^	7.022	0.698	0.850	0.008
ileum	230.183	223.912	217.078	244.630	7.016	0.792	0.464	0.248
colon	258.561 ^a^	243.451 ^ab^	233.565 ^b^	252.174 ^ab^	4.029	0.297	0.821	0.037
MDA (mmol/L; nmol/mg protein)								
plasma	1.735	1.725	2.114	1.939	0.070	0.036	0.494	0.539
duodenum	1.074	1.089	1.488	1.301	0.058	0.005	0.408	0.330
jejunum	0.48 ^a^	0.344 ^b^	0.417 ^ab^	0.431 ^ab^	0.018	0.718	0.068	0.028
ileum	1.264 ^a^	0.936 ^b^	1.383 ^a^	0.424 ^c^	0.082	0.029	0.000	0.001
colon	0.492 ^b^	0.378 ^b^	0.548 ^ab^	0.710 ^a^	0.037	0.005	0.698	0.036
GSH-PX (U/L; U/mg protein)								
plasma	276.408	299.433	275.274	276.327	3.975	0.112	0.114	0.148
duodenum	151.147 ^a^	112.024 ^b^	135.487 ^a^	151.521 ^a^	4.661	0.120	0.131	0.001
jejunum	83.686	99.663	96.985	103.581	3.150	0.161	0.070	0.438
ileum	179.803	172.545	129.562	113.191	7.046	0.000	0.229	0.638
colon	123.696	117.602	101.892	119.176	4.112	0.221	0.494	0.159
MPO (U/mL; U/mg protein)								
plasma	44.957	43.714	42.355	38.847	1.442	0.212	0.423	0.701
duodenum	0.154 ^c^	0.172 ^b^	0.189 ^a^	0.153 ^c^	0.018	0.054	0.027	0.000
jejunum	0.076 ^b^	0.088 ^ab^	0.101 ^a^	0.073 ^b^	0.004	0.438	0.206	0.005
ileum	0.194	0.188	0.227	0.185	0.005	0.111	0.012	0.058
colon	0.116	0.094	0.106	0.099	0.003	0.678	0.029	0.237

Values are means and pooled SEMs; *n* = 7. ^a,b,c^ Within a row, means with different superscripts differ; *p* < 0.05. H_2_O_2_: hydrogen peroxide; T-SOD: total superoxide and dismutase; MDA: malondialdehyde; GSH-Px: glutathione peroxidase; MPO: myeloperoxidase.

## Data Availability

All data generated or analyzed during this study are included in this published article. The data of the current study are available from the corresponding author upon request.
